# Efficacy of a novel self-expandable metal stent with dumbbell-shaped flare ends for distal biliary obstruction due to unresectable pancreatic cancer

**DOI:** 10.1038/s41598-022-25186-2

**Published:** 2022-12-06

**Authors:** Masaki Miyazawa, Hajime Takatori, Hirofumi Okafuji, Tomoyuki Hayashi, Tadashi Toyama, Shinya Yamada, Kazuya Kitamura, Kuniaki Arai, Yoshio Sakai, Taro Yamashita, Tatsuya Yamashita, Eishiro Mizukoshi, Masao Honda, Shuichi Kaneko

**Affiliations:** 1 Department of Gastroenterology, Kanazawa University Hospital, 13-1, Takara-Machi, Kanazawa City, Ishikawa Prefecture 920-8641 Japan; 2 Innovative Clinical Research Center, Kanazawa University, 13-1, Takara-Machi, Kanazawa City, Ishikawa Prefecture 920-8641 Japan

**Keywords:** Gastroenterology, Gastrointestinal diseases, Pancreatic disease, Pancreatic cancer

## Abstract

This study aimed to evaluate the efficacy of a novel fully covered self-expandable metal stent (SEMS) with dumbbell-shaped flare ends for the palliation of distal biliary obstruction (DBO) due to unresectable pancreatic cancer (UPC). Patients with DBO due to UPC who received the novel HILZO fully covered stent (HFS), the WALLFLEX partially covered stent (WPS) or fully covered stent (WFS) were analyzed. The incidence of recurrent biliary obstruction (RBO), time to RBO (TRBO), and the incidence of complications were compared among the three SEMS groups. Eighty-four patients (HFS, *n* = 36; WPS, *n* = 20; WFS, *n* = 28) were included. The incidence of RBO was low in the HFS group (versus the WPS and WFS group, *p* = 0.033 and 0.023, respectively). TRBO in the HFS group was longer than that in the WFS group (*p* = 0.049). Placement of the HFS was an independent factor for long TRBO in multivariable analysis (*p* = 0.040). The incidence of pancreatitis and cholecystitis in the HFS group was low (one for each). It is recommended to use the HFS for the palliation of DBO due to UPC from the viewpoint of the low incidence of RBO and complications.

## Introduction

Patients with distal biliary obstruction (DBO) due to unresectable pancreatic cancer (UPC) require adequate clinical palliation before chemotherapy. Self-expandable metal stent (SEMS) placement during endoscopic retrograde cholangiopancreatography (ERCP) is increasingly used because of its high effectiveness in relieving DBO^1,2^. However, recurrent biliary obstruction (RBO) associated with tumor ingrowth through the metal mesh remains a clinical problem^3–6^. To overcome tumor ingrowth, fully or partially covered SEMSs have been developed^7–9^, but concerns remain regarding the incidence of complications such as pancreatitis, cholecystitis, and stent migration^8,10–13^.

**Figure 1:**
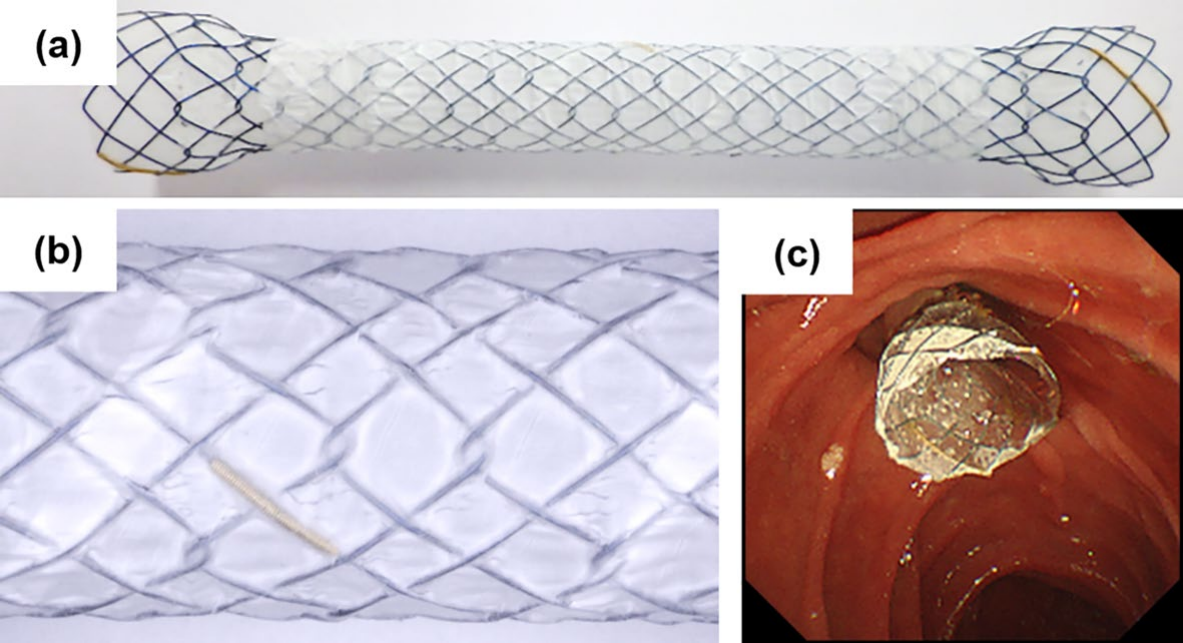
A novel fully covered SEMS with inner and outer PTFE coating and dumbbell-shaped flares. (**a**) Both the inside and outside of the metal mesh are coated with PTFE, and both ends of the stent have dumbbell-shaped flares. (**b**) The stent body has a nitinol-based braided hook-cross wire structure with a compositional ratio of cross and hook portions of 4–1. (**c**) The distal flare with a diameter 4 cm larger than that of the stent body allows anchoring to the duodenal papilla and reduces sludge accumulation. *EMS* Self-expandable metal stent, *PTFE* Polytetrafluoroethylene.

Currently, several types of covered SEMS are available depending on the individual case. They have different properties depending on the mesh material, the structure of the stent, the shape of ends of the stent, in addition to whether the stent membrane is fully or partially covered^11,14–16^. Recently, a novel fully covered SEMS (HILZO™), which have an inner and outer polytetrafluoroethylene (PTFE) coating and dumbbell-shaped flare ends, was approved for clinical use (BCM Co., Ltd, Paju, Korea). As shown in Figure 1, the HILZO™ fully covered stent (HFS) has a nitinol-based braided hook-cross wire structure, and both the inside and outside of the metal mesh are coated with PTFE. The impermeable surface and improved formability of the HFS are expected to reduce tumor ingrowth and stent migration compared to conventional silicone-coated covered SEMSs. Moreover, the dumbbell-shaped flares at both ends of the HFS should reduce the incidence of RBO induced by sludge accumulation and stent migration. Thus, in this study, we compared the HFS with conventional covered SEMSs and evaluated its efficacy and safety for the palliation of DBO in patients with UPC.

## Results

### Patient characteristics and SEMS placement

**Figure 2:**
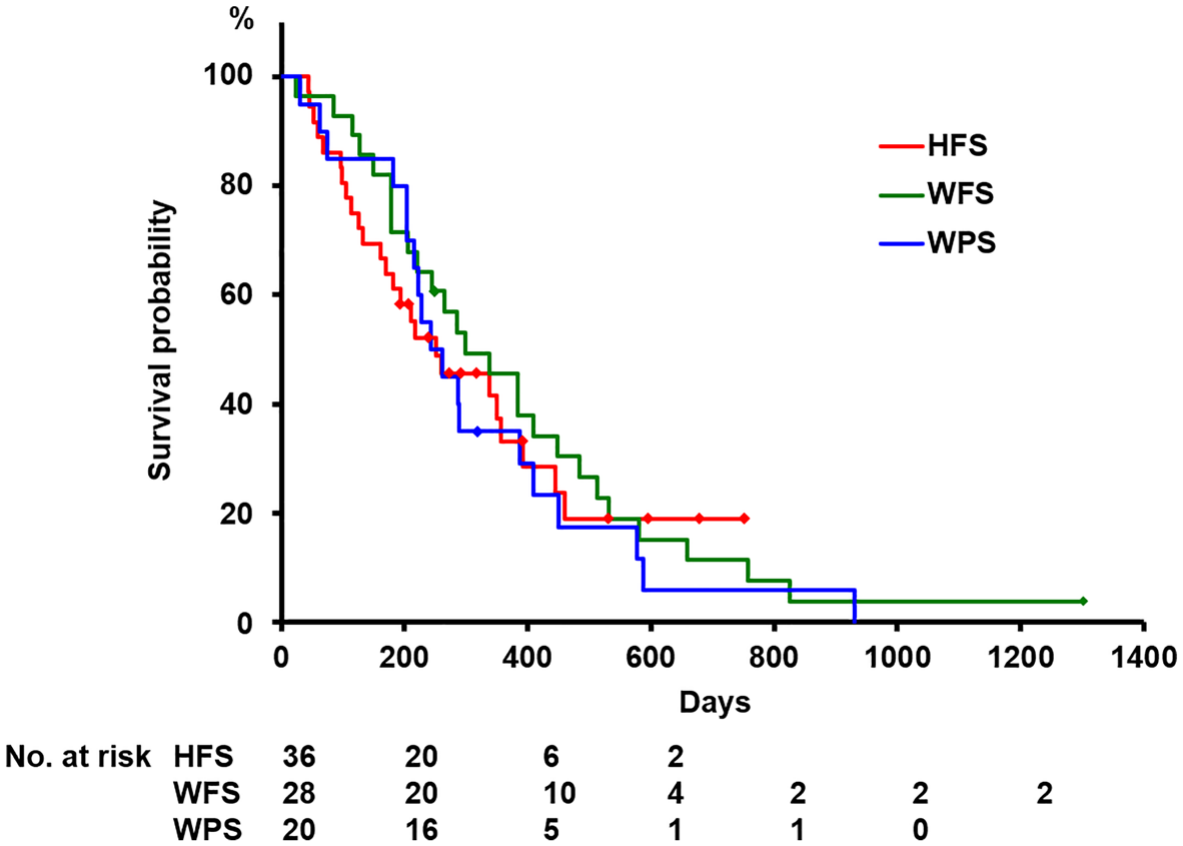
Kaplan–Meier plots for the estimation of the overall survival. There was no significant difference between the three groups in median survival time (251, 243 and 298 days in the HFS, WPS and WFS group, respectively, *p* = 0.785). *HFS* HILZO fully covered stent, *WPS* WALLFLEX biliary RX partially covered stent, *WFS* WALLFLEX biliary RX fully covered stent.

A total of 84 patients were enrolled in this study. Of these, 36 were received the HFS, 20 received the WPS, and 28 received the WFS. The characteristics of patients are summarized in Table 1. Significant variability was observed among the three groups in ascites (*p* = 0.020) and sphincterotomy before SEMS placement (*p* < 0.001). There was no significant difference between the three groups in age, sex, serum bilirubin level, serum ALP level, gallbladder stones / sludge, tumor involvement to the orifice of cystic duct, duodenal invasion, duodenal stent placement, length of the stricture, prior biliary drainage before SEMS placement, the length of the SEMS, and anti-cancer treatment. All patients enrolled in this study received a SEMS with 10 mm in the diameter. Technical and functional success was achieved in all patients. Kaplan–Meier plot of the overall survival is shown in Figure 2. There was no significant difference between the three groups in median survival time after SEMS placement (251, 243 and 298 days in the HFS, WPS and WFS group, respectively, *p* = 0.785).

**Table 1:** Clinical characteristics of enrolled patients.

	HFS (*n* = 36)	WPS (*n* = 20)	WFS (*n* = 28)	*p*-value
Age, years	72 (36–86)	69.5 (55–94)	66.5 (46–82)	0.280
Male gender	19 (52.8)	13 (46.4)	11 (55.0)	0.816
Bilirubin level, mg/dl	2.5 (0.8–12.8)	3.1 (0.9–20.2)	4.1 (0.8–32.0)	0.274
Alkaline phosphatase level, U/L	382.5 (156–2089)	370 (187–953)	307 (167–1710)	0.265
Gallbladder stones / sludge	3 (8.3)	2 (10.0)	5 (17.9)	0.484
Ascites	11 (30.6)	0 (0)	5 (17.9)	0.020
Tumor involvement to the orifice of cystic duct	2 (5.6)	0 (0)	3 (10.7)	0.300
Duodenal invasion	15 (41.7)	9 (45.0)	8 (28.6)	0.433
Duodenal stent placement	8 (22.2)	5 (25.0)	5 (17.9)	0.829
Prior biliary drainage before SEMS placement	25 (69.4)	10 (50.0)	18 (64.3)	0.348
Sphincterotomy before SEMS placement	30 (83.3)	4 (20.0)	22 (78.6)	< 0.001
Length of the stricture, mm	23 (12–50)	23 (10–36)	23 (11–37)	0.691
Diameter of the dilated bile duct, mm	11.9 (6.6–21.0)	12.4 (8.5–17.1)	12.1 (7.6–21.0)	0.497
The length of SEMS; 4 cm / 6 cm / 8 cm	0 (0) / 31 (86.1) / 5 (13.9)	3 (15.0) / 14 (70.0) / 3 (15.0)	4 (14.3) / 23 (82.1) / 1 (3.6)	0.104
Anti-cancer treatment after SEMS placement	30 (83.3)	18 (90.0)	28 (100)	0.079
Median survival time after SEMS placement, (range), days	251 (44–751)	243 (31–930)	298 (24–1032)	0.785

Data are presented as median (range) or number (proportion).

*SEMS* Self-expandable metal stent, *HFS* HILZO fully covered stent, *WPS* WALLFLEX biliary RX partially covered stent, *WFS* WALLFLEX biliary RX fully covered stent.

### RBO and other SEMS-related complications

**Figure 3:**
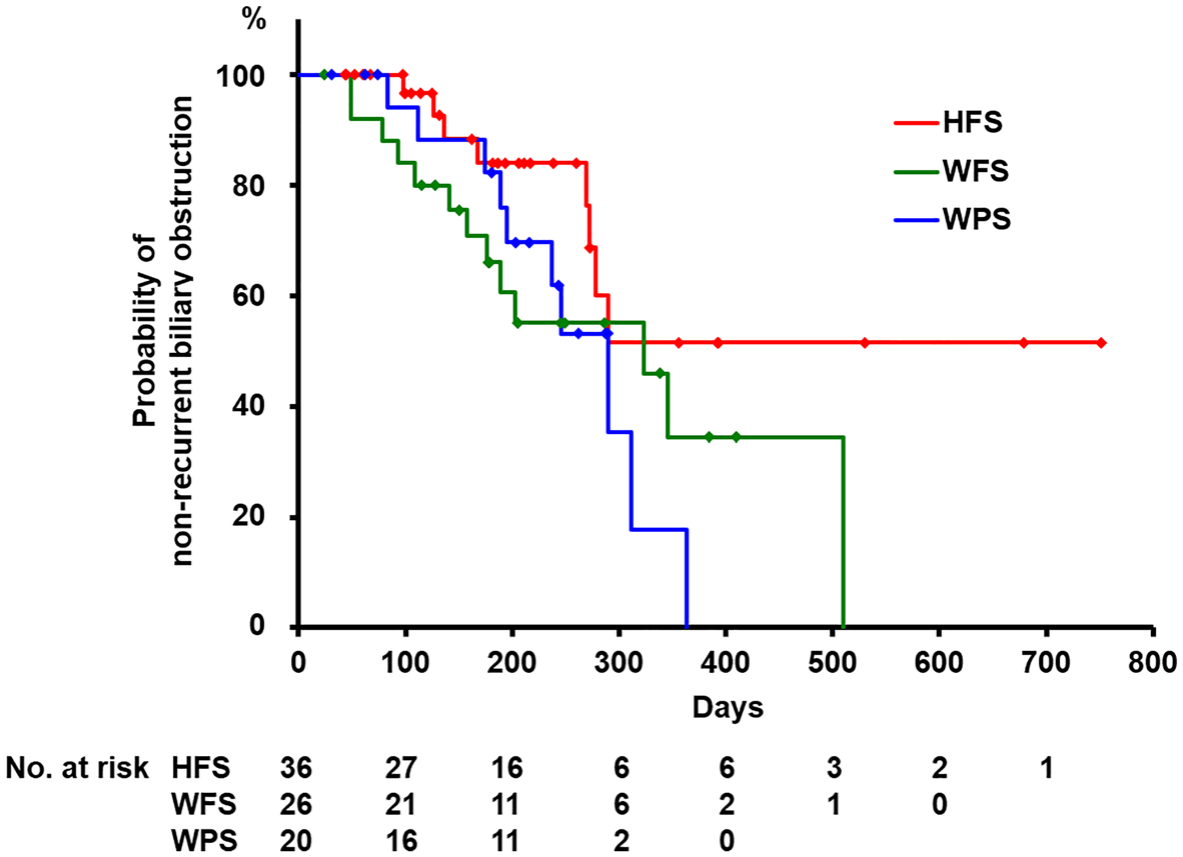
Kaplan–Meier plots for the estimation of the TRBO. Median TRBO was not reached in the HFS group, while 290 and 323 days in the WPS and WFS group, respectively (*p* = 0.108). TRBO of the HFS group was significantly longer than that of the WFS group (*p* = 0.049), and tended to be longer than that of the WPS group (*p* = 0.074). *TRBO* Time to recurrent biliary obstruction, *HFS* HILZO fully covered stent, *WPS* WALLFLEX biliary RX partially covered stent, *WFS* WALLFLEX biliary RX fully covered stent.

The incidences of RBO in each SEMS group are shown in Table 2. When comparing the RBO rate and TRBO between the three groups, we excluded two patients in the WFS group in which SEMS was removed due to complications other than RBO. During the follow–up period, the cumulative incidence of RBO was 22.2% in the HFS group, 50.0% in the WPS group, and 50.0% in the WFS group. Compared to the HFS group, the other two groups had a higher incidence of RBO (*p* = 0.033 and 0.023 in the WPS group and WFS group, respectively). Kaplan–Meier plot of the TRBO is shown in Figure 3. Median TRBO was not reached in the HFS group, while 290 and 323 days in the WPS and WFS group, respectively (*p* = 0.108). TRBO of the HFS group was significantly longer than that of the WFS group (*p* = 0.049), and tended to be longer than that of the WPS group (*p* = 0.074). The non-RBO rates at 3, 6, and 12 months estimated using the Kaplan–Meier method were 100%, 82.6% and 38.5% in the HFS group, 94.1%, 81.3%, and 0% in the WPS group, and 88.0%, 60.0%, and 20.0% in the WFS group, respectively. The non-RBO rates at 3, 6, 12 months in each SEMS group are shown in Supplementary Table S1 online. At 3 months after SEMS placement, the non-RBO rate was significantly higher in the HFS group than in the WFS group (*p* = 0.047). The non-RBO rate at 12 months of the HFS group was higher than that of the WPS group (*p* = 0.027).

**Table 2:** The incidences of recurrent biliary obstruction and its causes.

	HFS (*n* = 36)	WPS (*n* = 20)	WFS (*n* = 26)^a^	*p*-value
**Recurrent biliary obstruction (RBO)**				
Total	8 (22.2)	10 (50.0)	13 (50.0)	0.033 (HFS vs. WPS) 0.023 (HFS vs. WFS)
Sludge accumulation	1 (2.8)	6 (30.0)	3 (11.5)	0.003 (HFS vs. WPS) 0.166 (HFS vs. WFS)
Food impaction	2 (5.6)	0 (0)	2 (7.7)	0.471
Hemobilia	0 (0)	1 (5.0)	0 (0)	0.208
Tumor overgrowth	2 (5.6)	0 (0)	3 (11.5)	0.264
Tumor ingrowth /mucosal hyperplasia	0 (0)	2 (10.0)	0 (0)	0.053 (HFS vs. WPS)
Stent migration	3 (8.3)	1 (5.0)	5 (19.2)	0.246
Removal of SEMS	8 (22.2)	2 (10.0)	7 (25.0)	0.411

Data are presented as median (range) or number (proportion).

*SEMS* Self-expandable metal stent, *HFS* HILZO fully covered stent, *WPS* WALLFLEX biliary RX partially covered stent, *WFS* WALLFLEX biliary RX fully covered stent.

^a^Two patients in which SEMS was removed due to complications other than RBO were excluded.

The causes of RBO are shown in Table 2. The most common cause of RBO was stent occlusion, which occurred in 22 (26.8%) of all patients. Stent occlusion was mainly resulted from sludge accumulation and food impaction. Compared to the HFS group, the WPS group had a higher incidence of sludge accumulation (*p* = 0.003). The incidences of food impaction, tumor overgrowth and hemobilia were not significantly different between the three groups. No tumor ingrowth / mucosal hyperplasia occurred in the HFS group, while two (10.0%) patients developed it in the WPS group (*p* = 0.053). Stent migration was also a major cause of RBO. RBO-inducing stent migration occurred in 8.3% in the HFS group, 5.0% in the WPS group, and 19.2% in the WFS group (*p* = 0.246). Bile duct kinking did not contribute to RBO. SEMS removal was required in 15 patients (17.9%) and successful in all of them.

The univariable and multivariable analyses of the risk factors for RBO are shown in Table 3. Univariable analysis revealed that placement of the HFS is the most associated factor with a reduced incidence of RBO among several parameters; the HFS group had a low incidence of RBO compared to the WFS group (*p* = 0.023, HR 0.29, 95% CI 0.10–0.86). Age, gender, gallbladder stones / sludge, ascites, tumor involvement to the orifice of cystic duct, duodenal invasion, duodenal stent placement, prior biliary drainage before SEMS placement, sphincterotomy before SEMS placement, the length of the stricture, the diameter of the dilated bile duct, the length of the SEMS, and anti-cancer treatment were not associated with the incidence of RBO. The following six parameters were used as explanatory variables in the Cox proportional hazards model as potential risk factors for RBO: age, gender, gallbladder stones / sludge, duodenal invasion, the type of SEMS, and anti-cancer treatment. Placement of the HFS was the only independent factor for long TRBO (*p* = 0.040, HR 0.37, 95% CI 0.14–0.96).

**Table 3:** The incidences of recurrent biliary obstruction according to various clinical factors.

	*n* = 82^a^	RBO (n = 31)	Univariable		Multivariable	
			HR (95% CI)	*p*-value	HR (95% CI)	*p*-value
Age	< 70 years	17 (42.5)	1.00 (reference)		1.00 (reference)	
	≥ 70 years	14 (33.3)	0.68 (0.28–1.66)	0.392	0.75 (0.35–1.57)	0.442
Gender	Male	20 (46.5)	1.00 (reference)		1.00 (reference)	
	Female	11 (28.2)	0.45 (0.18–1.13)	0.088	0.56 (0.25–1.24)	0.150
Gallbladder stone / sludge	No	25 (34.7)	1.00 (reference)		1.00 (reference)	
	Yes	6 (60.0)	2.82 (0.73–10.93)	0.122	1.75 (0.66–4.63)	0.260
Ascites	No	27 (40.3)	1.00 (reference)			
	Yes	4 (26.7)	0.54 (0.16–1.87)	0.325		
Tumor involvement to the orifice of cystic duct	No	30 (38.0)	1.00 (reference)			
	Yes	1 (33.3)	0.82 (0.07–9.40)	0.871		
Duodenal invasion	No	16 (32.0)	1.00 (reference)		1.00 (reference)	
	Yes	15 (46.9)	1.86 (0.75–4.68)	0.175	1.77 (0.80–3.93)	0.159
Duodenal stent placement	No	23 (35.9)	1.00 (reference)			
	Yes	8 (44.4)	1.43 (0.49–4.12)	0.511		
Prior biliary drainage before SEMS placement	No	13 (41.9)	1.00 (reference)			
	Yes	18 (35.3)	0.76 (0.30–1.89)	0.548		
Sphincterotomy before SEMS placement	No	12 (42.9)	1.00 (reference)			
	Yes	19 (35.2)	0.72 (0.28–1.84)	0.497		
Length of the stricture	< 23 mm	12 (31.6)	1.00 (reference)			
	≥ 23 mm	19 (43.2)	1.65 (0.66–4.08)	0.280		
Diameter of the dilated bile duct	< 12 mm	14 (37.8)	1.00 (reference)			
	≥ 12 mm	17 (37.8)	1.00 (0.41–2.45)	0.996		
The type of SEMS	WFS	13 (50.0)	1.00 (reference)		1.00 (reference)	
	WPS	10 (50.0)	1.00 (0.31–3.21)	1.000	0.82 (0.31–2.18)	0.690
	HFS	8 (22.2)	0.29 (0.10–0.86)	0.023	0.37 (0.14–0.96)	0.040
The length of SEMS	4 cm	4 (57.1)	1.00 (reference)			
	6 cm	23 (34.8)	0.40 (0.08–1.95)	0.245		
	8 cm	4 (44.4)	0.60 (0.08–4.40)	0.614		
Anti-cancer treatment after SEMS placement	No	1 (12.5)	1.00 (reference)		1.00 (reference)	
	Yes	30 (40.5)	4.77 (0.56–40.81)	0.120	4.01 (0.45–35.68)	0.213

Data are presented as number (proportion).

*RBO* Recurrent biliary obstruction, *HR* Hazard ratio, *CI* Confidence interval, *SEMS* Self-expandable metal stent, *HFS* HILZO fully covered stent, *WPS* WALLFLEX biliary RX partially covered stent, *WFS* WALLFLEX biliary RX fully covered stent.

^a^Two patients in which SEMS was removed due to complications other than RBO were excluded.

The incidences of SEMS-related complications are shown in Table 4. The incidence of pancreatitis was not significantly different between the three groups, but tended to be slightly higher in the WFS group (*p* = 0.068). Of the patients who had pancreatitis, two of the WFS group required SEMS removal, while the others did not. The incidence of cholecystitis was calculated excluding patients after cholecystectomy and not significantly different between the three groups. Of the three patients who had cholecystitis, gallbladder stones / sludge and tumor involvement to the orifice of cystic duct was observed in one case each in the WFS group. The incidence of non-occlusion cholangitis was not significantly different between the three groups. All the patients with non-occlusion cholangitis improved after conservative treatment.

**Table 4:** The incidences of SEMS-related complications.

	HFS (*n* = 36)	WPS (*n* = 20)	WFS (*n* = 28)	*p*-value
**Complications after SEMS placement**				
Pancreatitis	1 (2.8)	0 (0)	4 (14.3)	0.068
Cholecystitis	1 (3.0)^a^	0 (0)^a^	2 (7.7)^a^	0.407
Non-occlusion cholangitis	11 (30.6)	3 (15.0)	6 (23.1)	0.436

Data are presented as median (range) or number (proportion).

*SEMS* Self-expandable metal stent, *HFS* HILZO fully covered stent, *WPS* WALLFLEX biliary RX partially covered stent, *WFS* WALLFLEX biliary RX fully covered stent.

^a^Patients who had undergone cholecystectomy were excluded.

## Discussion

In patients with malignant DBO, SEMSs are associated with a lower rate of RBO than plastic stents, such that the frequency of re-intervention will be reduced^2^. The 2019 Clinical Practice Guidelines for Pancreatic Cancer of the Japan Pancreas Society recommend SEMSs over plastic stents in patients with DBO in the treatment of UPC^17^. A longer TRBO and less frequent re-intervention are desirable because some intense chemotherapy regimens prolong patient survival.

Both covered and uncovered SEMSs are currently in use. Covered SEMSs were introduced to overcome tumor ingrowth, an disadvantage of uncovered SEMSs^7–9^. However, tumor ingrowth in covered SEMSs is unlikely, while stent migration is more frequently observed with these SEMSs. There is a controversy as to which of the covered and uncovered SEMSs is superior in terms of the resistance to RBO^8,10,11^. Since it is difficult to overcome the adverse consequences of tumor ingrowth in uncovered SEMSs, covered SEMSs resistant to migration are needed.

Various structural devices have been applied to covered SEMSs in order to reduce the risk of migration. Two mechanical properties of SEMS are deterministic factors that influence the risk of stent migration in DBO; the axial force (AF), defined as the force that restores the SEMS to its original straight shape after it has been bent during placement, and the radial force (RF), which is the expansion force against the stricture. SEMSs with a high AF and a low RF tend to cause stent migration frequently^18^. In recent years, several types of covered SEMSs with ideal mechanical characteristics such as a low AF and a high RF have been developed^19,20^. Furthermore, various anti-migration systems have been developed to overcome the slipperiness of covered SEMSs, including uncovered flared ends^11^, anchoring fins^21^, and raised bands on the stent body^15^. Partially covered SEMSs have uncovered ~5-mm sections on either end, which are expected to reduce the risk of stent migration. There are some reports comparing the WFS and the WPS. A retrospective study reported that the incidence of stent migration was significantly higher in the WFS group than that in the WPS group^16^. However, a prospective multicenter study comparing them reported no significant difference in the rate of stent migration between two groups in patients with malignant DBO^22^. It is unclear whether the fully or partially covered SEMS is suitable for the palliation of malignant DBO. Since it has been shown that tumor shrinkage caused by anti-cancer treatment is likely to cause stent migration^18^, a high RF and effective anti-migration system are especially important properties for patients with DBO due to UPC.

The HILZO™ fully covered stent (HFS) has an inner and outer PTFE coating and dumbbell-shaped flares. The body of the novel SEMS has a nitinol-based braided hook-cross wire structure with a compositional ratio of cross and hook portions of 4 to 1. Among braided SEMSs, those with only a cross wire structure have a high AF that can cause shortening, bile duct kinking, sludge accumulation, and stent migration^9^. However, SEMSs with only a hook wire structure have a low RF and are thus also vulnerable to migration^18^. Although details of the AF and RF of the HFS have not been released by the manufacturer, both likely fall between the values of the cross wire types and hook wire types, such that a low AF and moderate RF can be expected. Furthermore, the inside and outside parts of the metal mesh are coated with PTFE (Teflon^TM^), which has a low coefficient of friction. As a result, the inner coating of the HFS appears to more effectively prevent sludge accumulation and food impaction in the stent lumen than standard silicone-coated SEMSs like the WPS and the WFS. In addition, the outer coating has high biocompatibility with the bile duct wall and its impermeable surface strongly resists tumor ingrowth. Although PTFE-coated SEMSs have been used from before^21,23^, the inner and outer coating of the HFS is unique. Moreover, an 1-cm flare on both ends of the HFS, which accounts for its dumbbell shape, has a diameter 4 mm larger than that of the stent body, and their silicone coating counteracts the slipperiness of the PTFE-coated stent body. The larger caliber of the distal flared end also allows anchoring to the duodenal papilla and reduces sludge accumulation.

This is the first clinical study comparing the performance of the novel HFS with that of other types of covered SEMSs, namely the WPS and the WFS, in patients with DBO due to UPC. During the follow-up periods, in which there was no significant difference between the three SEMS groups, the cumulative incidence of RBO in the HFS group was significantly lower than that in the other two groups. Compared to the HFS group, TRBO was significantly shorter in the WFS group, and the non-RBO rate at 12 months was lower in the WPS group. In previous studies evaluating the performance of different types of fully covered SEMSs, the median TRBO was 153–373 days^11,12,14,16,20,22^. Although it may be inappropriate to unconditionally compare the median TRBO of the HFS with the results of past studies due to the difference in clinical backgrounds, it is considered that our result is not inferior to them. It is notable that despite variations in clinical parameters (e.g. ascites and sphincterotomy) between the three SEMS groups in this study, the only factor contributing to long TRBO in multivariable analysis was placement of the HFS.

There were some differences in the cause of RBO between the three SEMS groups. The rate of RBO-inducing stent migration tended to be slightly higher in the WFS group, while there was almost no stent migration in the WPS group, probably because of the uncovered section on either end of the SEMS. The frequency of stent migration was also relatively low in the HFS group, which may be the effect of a low AF and dumbbell-shaped flare ends. Sludge accumulation and tumor ingrowth /mucosal hyperplasia were main causes of RBO in the WPS group and likely to occur in the uncovered section on either end of the SEMS. The low incidence of sludge accumulation in the HFS group may have been caused by an inner coating with PTFE and a larger caliber of the distal flared end. Although our study couldn't identify the decisive reason why the HFS group had a longer TRBO than the WFS group, the slightly lower incidence of stent migration and sludge accumulation seemed to lead to better results for the HFS group.

The incidence of SEMS-related complications other than RBO, such as pancreatitis and cholecystitis, may increase with the increased opportunity to use covered SEMSs, as these stents compress the orifice of the pancreatic duct and cystic duct, respectively. In UPC patients, dilation of the pancreatic duct is thought to reduce the risk of pancreatitis even if a fully covered SEMS was placed^24^. In our study, there was no difference in the incidence of pancreatitis between the three types of SEMS. However, compared to the HFS group, the WFS group tended to develop pancreatitis more often. It has been reported that SEMS with a high AF was associated with a high incidence of pancreatitis in patients with malignant DBO other than UPC^25^. Similar results were obtained in our study which enrolled UPC patients only, and it is considered that a high AF of the WFS, which is a braided SEMS consisting of only a cross wire structure, makes pancreatitis more likely to occur. None of patients in the WPS group developed pancreatitis, probably because the uncovered section on the end of the WPS did not occlude the orifice of the pancreatic duct. Although the incidence of cholecystitis was not significantly different between the three groups, no patient developed cholecystitis in the WPS group, probably because the uncovered section on proximal end of the SEMS did not compress too hard the orifice of the cystic duct. Previous studies have reported that gallbladder stones, the tumor involvement of the orifice of the cystic duct, and the use of covered SEMS with a high AF were risk factors for cholecystitis after SEMS placement^13,26^. Of the three patients who had cholecystitis in our study, gallbladder stones / sludge and tumor involvement to the orifice of cystic duct was observed in one case each in the WFS group. Although the HFS is expected to have a low AF, its dumbbell-shaped flares may increase the risk of cholecystitis due to pressure on the orifice of the cystic duct. We should be careful about the development of cholecystitis in patients who need placement of the HFS such that it reaches the confluence of the cystic duct and common hepatic duct.

The limitations of our study included its retrospective design and the recruitment of patients from a single institution. Although each SEMS group consisted of consecutive UPC patients who received a SEMS for the palliation of DBO, patients in the HFS group and the other two groups did not undergo SEMS placement within the same period, suggesting that there are biases that affect TRBO and complications, such as patients’ characteristics, endoscopic procedures including sphincterotomy and SEMS placement, and anti-cancer treatment after SEMS placement. Similarly, there may have been a selection bias between the WFS and WPS groups. It is desirable to enroll sufficient patients in a prospective study in order to eliminate bias as much as possible. Furthermore, multicenter randomized controlled trials comparing other types of covered SEMS are necessary to confirm the true efficacy and safety of the HFS.

In conclusion, the HFS demonstrated a low incidence of RBO with a low risk of SEMS-related complications. Therefore, the HFS can be recommended for the palliation of DBO due to UPC.

## Methods

### Patients

This retrospective study included patients who received the HFS for the palliation of DBO due to UPC between January 2019, when the HFS began to be used, and June 2021. As historical controls, patients who received conventional covered SEMS: either the Wallflex™ biliary RX partially covered stent (WPS) or fully covered stent (WFS; Boston Scientific Corp., Marlborough, MA, USA) between January 2010 and December 2018 were enrolled as control groups in this study. In principle, each of three groups included consecutive UPC patients who received a SEMS during the above periods, but patients who met the following criteria were excluded from the subjects: previous SEMS placement or surgically altered anatomy previously, tumor invasion to the duodenal papilla, and a follow-up period of < 3 months (except in cases of death). Patients who underwent other biliary drainage because the endoscopist determined that SEMS placement was inappropriate for some reason were also excluded from this study. The diagnosis of UPC was based on the radiological and pathological examinations. Patients who needed urgent biliary drainage before SEMS placement underwent either biliary drainage with a plastic stent or endoscopic naso-biliary drainage (ENBD) as a bridge treatment to improve cholestasis. Even if serum bilirubin level was within the normal range, patients with both the elevation of serum alkaline phosphatase (ALP) level and the dilated bile duct on the image were considered to be at high risk of developing cholangitis during chemotherapy, so SEMS placement was performed on such patients. This study was conducted in accordance with the Declaration of Helsinki and approved by the Institutional Review Board of Kanazawa University Hospital (the approval number is 2020-824). This study involved personal data that were previously collected and did not require additional recruitment of human subjects; thus, a waiver of written informed consent was granted via the opt-out method by Ethics Committee of Kanazawa University Hospital. We disclosed the content of this study on the hospital’s website and provided an opportunity for patients to refuse to participate in this study.

### Endoscopic SEMS placement

In this study, all the SEMS placement procedures were performed by Board Certified Fellows of the Japan Gastroenterological Endoscopy Society with at least eight years of experience. All patients underwent transpapillary SEMS placement across the duodenal papilla during ERCP using a duodenoscope (JF-260V, TJF-260V and TJF-Q290V; Olympus Medical Systems, Tokyo, Japan) under conscious sedation with midazolam. Our institute has a policy of performing sphincterotomy when a fully-covered SEMS is placed in principle, although sphincterotomy may not be possible due to anatomical or patient background factors. On the other hand, because of the uncovered section on a distal end of the WPS, we thought that the risk of obstructing the orifice of the pancreatic duct was low, so sphincterotomy was not performed before placement of the WPS. Regarding the choice of the WFS or the WPS used at the same period, the WFS was placed when the tumor obstruction was located near the duodenal papilla, and the WPS was placed in other cases. The diameter and length of the SEMS were determined based on the diameter of the dilated bile duct, the length of the stricture, and other anatomical factors. Patients were routinely given prophylactic antibiotics and protease inhibitors before and after ERCP.

### Definitions and outcome measurements

Since April 2020, serum ALP level has been measured by the International Federation of Clinical Chemistry and Laboratory Medicine (IFCC) method. For serum ALP level measured by the Japan Society of Clinical Chemistry (JSCC) method before that, the regression formula for ALP value using the JSCC (*x*) and IFCC (*y*) methods was defined as *y* = 0.337*x* + 2.959 and *y* value was described as serum ALP level^27^.

Our main objective was to compare the incidence of recurrent biliary obstruction (RBO), time to RBO (TRBO), and the incidence of SEMS-related complications among the three SEMS groups. The terminology for transpapillary SEMS placement used in this study generally followed the TOKYO criteria 2014^28^. Technical success was defined as successful SEMS placement with sufficient coverage of the stricture as intended. Technical failure was defined as anything other than the above, including abandonment of SEMS placement after inserting a delivery system of SEMS into the instrument channel opening of the endoscope. Functional success was defined as a condition that met one of the following within 14 days: (1) > 50% decrease or normalization of serum bilirubin level, (2) > 50% decrease or normalization of serum ALP level if serum bilirubin level was within the normal range. If prior biliary drainage had been performed, functional success was defined as the achievement of the above condition after SEMS placement compared to the condition before the initial drainage. RBO was defined as a composite endpoint of either stent occlusion or migration with both clinical features suggestive biochemical evidence of cholestasis; i.e. elevated serum ALP level compared with baseline values, accompanied by insufficient biliary luminal patency on radiological images. The presence of stent occlusion or migration causing RBO was confirmed during a subsequent ERCP. The cause of stent occlusion was classified as follows: tumor ingrowth/overgrowth, mucosal hyperplasia, sludge accumulation, food impaction, hemobilia, bile duct kinking, or other. Since mucosal hyperplasia cannot be distinguished from tumor ingrowth despite biopsies or cholangioscopy occasionally, the two were treated indistinguishably. Stent migration sometimes induces RBO, while asymptomatic stent migration can also occur. When asymptomatic stent migration was detected incidentally, the SEMS was removed if it remained in the bile duct; then, biliary luminal patency was confirmed by cholangiography. These cases were defined as non-RBO according to the TOKYO criteria 2014^28^. Time to RBO (TRBO) was defined as the time from initial SEMS placement to the occurrence of RBO. In the estimation of TRBO, patient death, last visit, and SEMS removal due to complications other than RBO were classified as censored cases at the time of death, last visit, and SEMS removal, respectively. SEMS-related complications were also described according to the TOKYO criteria 2014^28^. Survival time was defined as the time from SEMS placement to patient death or last visit.

### Statistical analysis

IBM SPSS Statistics version 20.0 (IBM Corp., Armonk, NY, USA) was used for the statistical analyses. Continuous variables were analyzed using the Mann-Whitney U test for comparisons between the two groups and the Kruskal–Wallis test for comparisons between the three groups. Categorical variables were compared using the chi-squared test. TRBO and survival time were plotted using the Kaplan–Meier method and compared using the log–rank test. The Cox proportional hazards model was used to identify variables that affected differences in TRBO and estimate an adjusted hazard ratio (HR) with 95% confidence intervals (CIs). In this multivariable analysis, explanatory variables input to the analysis were selected with reference to previous studies^18,29,30^. Statistical significance was defined as a *p*-value < 0.05.

## Supplementary Information

Supplementary Information.

## Data Availability

Deidentified individual participant data are available and will be provided on reasonable request to the corresponding author.
