# Supplementary material for: Efficacy of a novel self-expandable metal stent with dumbbell-shaped flare ends for distal biliary obstruction due to unresectable pancreatic cancer
Source: Sci Rep. 2022 Dec 6;12:21100. doi: 10.1038/s41598-022-25186-2 (PMC9727129; doi:10.1038/s41598-022-25186-2)
Supplement: Supplementary file 1 — Supplementary Information. [file 41598_2022_25186_MOESM1_ESM.pdf]

Supplementary Table S1 Non-RBO rates at 3, 6 and 12 months in each SEMS group.

Non-RBO rate	HFS		WPS		WFS		*p*-value
	% (*n*)	95% CI	% (*n*)	95% CI	% (*n*)	95% CI	
3 months	100 (31/31)	N/A	94.1 (16/17)	71.3-99.9	88.0 (22/25)	68.8-97.5	0.172 (HFS vs. WPS)<br>0.047 (HFS vs. WFS)
6 months	82.6 (19/23)	61.2-95.1	81.3 (13/16)	54.4-96.0	60.0 (12/20)	36.1-80.9	0.913 (HFS vs. WPS)<br>0.099 (HFS vs. WFS)
12 months	38.5 (5/13)	13.9-68.4	0 (0/10)	N/A	20.0 (3/15)	4.3-48.1	0.027 (HFS vs. WPS)<br>0.281 (HFS vs. WFS)

Abbreviations: RBO, Recurrent biliary obstruction; SEMS, self-expandable metal stent; HFS, HILZO™ fully covered stent; WPS, Wallflex™ biliary RX partially covered stent; WFS, Wallflex™ biliary RX fully covered stent; CI, Confidence interval; NA, not available.
